# A software tool for stitching two PET/CT body segments into a single whole‐body image set

**DOI:** 10.1120/jacmp.v13i3.3599

**Published:** 2012-05-10

**Authors:** Tingting Chang, Guoping Chang, John W. Clark, Eric M. Rohren, Osama R. Mawlawi

**Affiliations:** ^1^ Department of Electrical and Computer Engineering Rice University Houston TX; ^2^ Department of Nuclear Medicine The University of Texas MD Anderson Cancer Center Houston TX; ^3^ Department of Imaging Physics The University of Texas MD Anderson Cancer Center Houston TX USA

**Keywords:** PET/CT, whole‐body imaging, stitching

## Abstract

A whole‐body PET/CT scan extending from the vertex of the head to the toes of the patient is not feasible on a number of commercially available PET/CT scanners due to a limitation in the extent of bed travel on these systems. In such cases, the PET scan has to be divided into two parts: one covering the upper body segment, while the other covering the lower body segment. The aim of this paper is to describe and evaluate, using phantom and patient studies, a software tool that was developed to stitch two body segments and output a single whole‐body image set, thereby facilitating the interpretation of whole‐body PET scans. A mathematical model was first developed to stitch images from two body segments using three landmarks. The model calculates the relative positions of the landmarks on the two segments and then generates a rigid transformation that aligns these landmarks on the two segments. A software tool was written to implement this model while correcting for radioactive decay between the two body segments, and output a single DICOM whole‐body image set with all the necessary tags. One phantom, and six patient studies were conducted to evaluate the performance of the software. In these studies, six radio‐opaque markers (BBs) were used as landmarks (three on each leg). All studies were acquired in two body segments with BBs placed in the overlap region of the two segments. The PET/CT images of each segment were then stitched using the software tool to create a single DICOM whole‐body PET/CT image. Evaluation of the stitching tool was based on visual inspection, consistency of radiotracer uptake in the two segments, and ability to display the resultant DICOM image set on two independent workstations. The software tool successfully stitched the two segments of the phantom image, and generated a single whole‐body DICOM PET/ CT image set that had the correct alignment and activity concentration throughout the image. The stitched images were viewed by two independent workstations from two different manufacturers, attesting the ability of the software tool to produce a DICOM compliant image set. The study demonstrated that this software tool allows the stitching of two segments of a whole‐body PET/CT scan with minimal user interaction, thereby facilitating the interpretation of whole body PET/CT scans from a number of scanners with limited extent of bed travel.

PACS number: 87.57.N, 87.57.uk

## I. INTRODUCTION

In PET/CT imaging, some protocols require the acquisition of whole‐body scans that cover the area from the top of the head to the bottom of the feet. Such scans, however, are often acquired as two separate imaging sessions due to a limitation in the extent of bed travel on some commercially available PET/CT systems. For example, the GE Discovery RX and STE scanners (GE Healthcare, Waukesha, WI) have a bed travel limited to 160 centimeters and cannot be used to image tall patients (> 160 cm) in a single imaging session. In such cases, a whole‐body PET/CT scan has to be divided into two acquisitions covering an upper and a lower body segment, respectively. For the upper body acquisition, the patient lies supine on the scanner couch while data is acquired using a head in or head out protocol. The patient is then removed from the scanner and flipped such that the feet are now pointing towards the scanner. Data acquisition is then performed to image the lower body segment of the patient. Image sets acquired in this manner make the display and interpretation difficult since each segment has to be evaluated separately. At our institution, patients imaged in this manner constitute about 20% of our overall PET/CT patient studies. This number is expected to increase in the future with the rising interest in PET/CT for bone imaging using NaF, since this type of scan requires an imaging extent that covers the top of the head to the bottom of the feet.

There are several disadvantages of acquiring two separate data sets for a whole‐body scan: 1) it reduces the physician's reading efficiency since two image sets need to be displayed rather than a single image set; 2) it requires the comparison of a patient's current and prior scans to be performed in two separate paired segments rather than a single session; 3) it does not allow the rendering of a patient's whole‐body image, hence cannot provide the radiologist with the ability to generate whole‐body images or maximum intensity projection (MIP).

There are several techniques to stitch two images in general. Zomet et al.^(^ ^1^ ^)^ introduced techniques to minimize the seam artifact and overcome both photometric inconsistencies and geometric misalignments between the stitched images. Brown and Lowe^(^ ^2^ ^)^ used invariant local features to find matches between the images. Such a technique is insensitive to the ordering, orientation, scale, and illumination of the input images and allows fully‐automated panoramic image stitching. More recently, image stitching for deformable structures has been investigated.^(^ ^3^ ^)^ This approach was based on structure deformation and propagation for achieving the overall consistency in image structure and intensity. Most of these general image stitching techniques, however, focused on 2D image stitching and assumed that the original image is of high resolution. In PET/CT imaging, both the PET and CT images of the two segments are 3D images with a small overlap region (about 5 cm), and the PET image is characterized by low image resolution.

The proposed software tool discussed in this paper utilizes simple mathematical techniques to perform the stitching task. The value of this tool is not in its introduction of new image manipulation techniques but lies primarily in its ability to stitch PET/CT images from two body segments with minimal user interaction, thereby overcoming the above disadvantages while at the same time facilitating the interpretation of whole‐body PET/CT studies. To our knowledge, no such software tool currently exists.

## II. MATERIALS AND METHODS

### A. Mathematical model

Current whole‐body PET/CT imaging protocols that require an imaging extent of > 160 cm are acquired in two imaging sessions on some scanners corresponding to an upper and lower body imaging segments. These two imaging segments are acquired with an overlapping area between the end of the first imaging session and the beginning of the second imaging session. This overlap ensures that the whole body of the patient is imaged without any anatomical truncation. Our proposed stitching method makes use of this overlap section to facilitate the stitching of the two body segments to one another.

**Figure 1:**
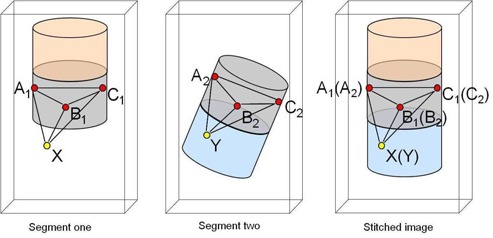
Illustration of the stitching method.

Consider three independent landmarks positioned in the overlap section of the two segments. The location of these landmarks are $\vec{A_{1}}$, $\vec{B_{1}}$, $\vec{C_{1}}$ in segment one, and $\vec{A_{2}}$, $\vec{B_{2}}$, $\vec{C_{2}}$ in segment two (Fig. 1), where the location of each landmark is represented by three coordinates (x, y, z). The overlap section is illustrated in gray in Fig. 1. To stitch these two segments, the positions of $\vec{A_{2}}$, $\vec{B_{2}}$, $\vec{C_{2}}$ should be re‐orientated such that they match $\vec{A_{1}}$, $\vec{B_{1}}$, $\vec{C_{1}}$, respectively, in the stitched image in Fig. 1. Consequently, a point $\vec{Y}$ in segment two will match an unknown point $\vec{X}$ in segment one. Stated differently, this problem can be viewed as finding the coordinates of unknown voxels $\vec{X}$ that correspond to each voxel $\vec{Y}$ in segment two. Knowing the voxel coordinates of $\vec{Y}$, ($\vec{A_{1}}$, $\vec{B_{1}}$, $\vec{C_{1}}$) and ($\vec{A_{2}}$, $\vec{B_{2}}$, $\vec{C_{2}}$), one can calculate the unknown coordinates of voxel $\vec{X}$ based on the assumption that the two body segments are characterized by rigid motion whereby the relative position of the landmarks remains unchanged between the image sets of the two body segments. This assumption is valid only if we assume the leg as a rigid body and the landmarks in the overlap region are located above or below the knee area; otherwise the lower/upper legs (shins/thighs) can articulate around the knee without a corresponding landmark movement. Three equations can be written to calculate the coordinates of voxel $\vec{X}$ according to the geometric relationships:

(1) $$\{\begin{array}{l} \left({\vec{A}}_{2}-{\vec{B}}_{2})\cdot (\vec{Y}-{\vec{B}}_{2})=({\vec{A}}_{1}-{\vec{B}}_{1})\cdot (\vec{X}-{\vec{B}}_{1}\right)\\ \left({\vec{C}}_{2}-{\vec{B}}_{2})\cdot (\vec{Y}-{\vec{B}}_{2})=({\vec{C}}_{1}-{\vec{B}}_{1})\cdot (\vec{X}-{\vec{B}}_{1}\right)\\ \left({\vec{A}}_{2}-{\vec{B}}_{2})\times ({\vec{C}}_{2}-{\vec{B}}_{2})\cdot (\vec{Y}-{\vec{B}}_{2})=({\vec{A}}_{1}-{\vec{B}}_{1})\times ({\vec{C}}_{1}-{\vec{B}}_{1})\cdot (\vec{X}-{\vec{B}}_{1}\right) \end{array}$$

The first equation (top line) indicates that the relative positions between $\vec{A_{2}},\vec{B_{2},}$, and $\vec{Y}$ remain the same as that between $\vec{A_{1}},\vec{B_{1},}$, and $\vec{X}$ in the images of the two body segments. Similarly, the second equation (middle line) indicates that the relative positions between $\vec{C_{2}},\vec{B_{2},}$, and $\vec{Y}$ remain the same as that of $\vec{C_{1}},\vec{B_{1},}$, and $\vec{X}$ also in the images of the two body segments. The third equation (last line), on the other hand, indicates that the volume of the pyramid $\vec{A_{2}},\vec{B_{2},}\vec{C_{2}}$, and $\vec{Y}$ is equal to the volume of the pyramid $\vec{A_{1}},\vec{B_{1},}\vec{C_{1}}$, and $\vec{X}$. Using this set of three equations in three unknowns, the coordinates of vector $\vec{X}$ can be determined.

Equation (1) can be rewritten as a matrix vector product, as shown in Eq. (2).

(2) $$\left(\begin{array}{l} \left({\vec{A}}_{2}-{\vec{B}}_{2})\cdot (\vec{Y}-{\vec{B}}_{2}\right)\\ \left({\vec{C}}_{2}-{\vec{B}}_{2})\cdot (\vec{Y}-{\vec{B}}_{2}\right)\\ \left({\vec{A}}_{2}-{\vec{B}}_{2})\times ({\vec{C}}_{2}-{\vec{B}}_{2})\cdot (\vec{Y}-{\vec{B}}_{2}\right) \end{array}\right)=\left(\begin{array}{l} {\left({\vec{A}}_{1}-{\vec{B}}_{1}\right)}^{T}\\ {\left({\vec{C}}_{1}-{\vec{B}}_{1}\right)}^{T}\\ {\left({\vec{A}}_{1}-{\vec{B}}_{1}\right)}^{T}\times {\left({\vec{C}}_{1}-{\vec{B}}_{1}\right)}^{T} \end{array}\right)\cdot (\vec{X}-{\vec{B}}_{1})$$

By carefully examining the first part of the right hand side of Eq. (2), one can see that $\vec{A_{1,}}\vec{B_{1}}$, and $\vec{C_{1}}$ cannot lie along the same line, otherwise the module of this matrix will be equal to 0, and Eq. (2) will have infinite solutions. This prerequisite is described by the inequality shown in Eq. (3) which, when enforced, will result in the existence and uniqueness of $\vec{X}$.

(3) $$\left|\begin{array}{l} {\left({\vec{A}}_{1}-{\vec{B}}_{1}\right)}^{T}\\ {\left({\vec{C}}_{1}-{\vec{B}}_{1}\right)}^{T}\\ {\left({\vec{A}}_{1}-{\vec{B}}_{1}\right)}^{T}\times {\left({\vec{C}}_{1}-{\vec{B}}_{1}\right)}^{T} \end{array}\right|\neq 0$$

Having determined the coordinates of $\vec{X}$, the value of voxel $\vec{X}$ can then be determined from the corresponding value of $\vec{Y}$, since $\vec{X}$ and $\vec{Y}$ are matched in the stitched image, as shown in Fig. 1.

**Figure 2:**
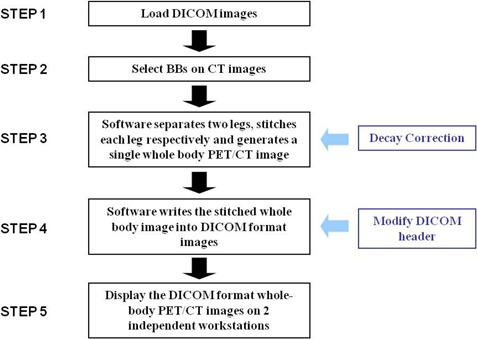
Flowchart of the stitching software.

**Figure 3:**
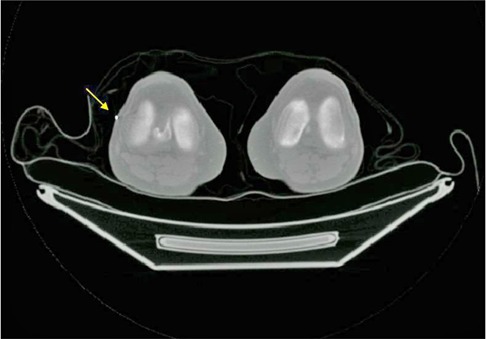
A typical CT image in transaxial FOV showing patient's two legs with a BB.

A flowchart of the stitching software algorithm is shown in Fig. 2. In step 1 of the flowchart, the two PET/CT image segments are loaded into the stitching program. In step 2, the positions of the landmarks in each segment are determined. Radio‐opaque markers such as BBs (2 mm pellets with easy pick‐up tab made by Beekley Corporation, Bristol, CT) were used as landmarks in the phantom and clinical studies to evaluate this method. These landmarks are very easy to segment on CT images since their Hounsfield units (CT number) are relatively very high. To determine the location of the BBs, the user has to manually select each BB by clicking on it on the software interface from three views of the CT images (Fig. 3). In the third step, the above mathematical model is applied to stitch the PET/CT images. For the phantom and clinical applications however, the images of the lower segment are first automatically split into two parts, each representing one leg of the patient prior to the stitching process. The purpose of the automated splitting function is to accommodate the possibility that patients might have moved their legs independently between the upper and lower imaging segments. The automatic splitting process is facilitated by relying on the different Hounsfield unit values between air and the patient legs. The software detects the smallest voxel value between the two legs and sets that voxel to be the boundary between the two legs. After stitching each leg respectively, the “black space” between the legs is filled with values of air (−1000 for CT and 0 for PET). In the fourth step, the stitched image set is written out as a DICOM object. Decay correction and DICOM header modification are essential for the resultant DICOM image set to have the proper tags and scaling factors. Decay correction is performed based on the time difference between the two imaging segments, and is implemented to correct the activity concentration in the second PET segment to the beginning of the first PET segment. The details in DICOM header modification are described in the next paragraph. Finally in step 5, the stitched DICOM whole‐body PET/CT images are ready for display. This five‐step algorithm was written as a software tool using MATLAB (The MathWorks, Natick, MA), and was used in the stitching of the phantom and clinical studies.

The tags in the DICOM header are essential for the proper display of the resultant DICOM image on various workstations. Therefore, several tags need to be modified in the stitched image in step 4. The parameters that needed to be edited are: *SliceLocation, InstanceNumber, ImageIndex, NumberofSlices, RescaleSlope, acquisition time*, and *series description*. The first three parameters are associated with the position or the order of each slice and have to be modified in order to allow a smooth transition between the two segments. The *NumberofSlices*, on the other hand, should reflect the sum of the slices of the two segments. The *RescaleSlope* has to be modified to incorporate the effect of decay correction on the second segment, while *acquisition time* of the second segment is modified to be the time of the first segment, since decay correction has already been applied to the stitched images. Finally, the *series description* is set to be consistent for all the slices when saving the stitched DICOM.

### B. Phantom study

A phantom study was first conducted to evaluate the performance of the software tool. Two 1.5 liter bottles were used to simulate two legs of a patient. The bottles were filled with ${\;}^{18}F$ water with an activity concentration of 3.33 kBq/cc(0.09 μCi/cc) to emulate the common background activity concentration in patients. Three BBs were attached to each bottle at the planned overlap section before the scans. Data acquisition was performed using a GE Discovery RX PET/CT scanner.^(^ ^4^ ^)^ The first imaging segment consisted of a PET/CT scan of 3 min duration in 3D mode, which is the standard clinical PET/CT imaging parameter at our institution. After the first scan, the bottles were flipped and re‐orientated to simulate a patient moving their legs between the first and second imaging segments. In the second imaging segment, the same imaging sequence was repeated after a delay period of 30 min. This delay period ensured a discrepancy in the amount of radioactivity between the two segments and tested the software's ability to correct for the differences in activity concentration between the two imaging segments. For both segments, the CT images were used for attenuation correction. The CT protocol was 120 kVp, 150 mAs, pitch 1.375, slice thickness of 3.75, and interval of 3.27 mm. The PET data were reconstructed using OSEM (2 iterations, 21 subsets) and a postreconstruction filter (6 mm) was applied, which is the standard clinical protocol in our institution.

After the acquisition, the software tool was used to stitch the two segments of PET/CT images. Visual inspection was used to evaluate the accuracy of the stitching process by assessing the presence of any discontinuity at the stitching plane. In addition, the accuracy of radiotracer concentration in the stitched PET image set was assessed by plotting a line profile along the axial direction of the stitched PET dataset and evaluating the consistency of the activity concentration in that direction. Furthermore, the line profiles in the overlap area from the prestitched image were also included for comparison between prestitched and stitched image in the overlap region. In each axial slice, the activity concentration was determined as the mean of 50 pixels in a region of interest within that slice. The ability to display the resultant DICOM image set on two independent workstations (GE Advantage (GE Healthcare, Waukesha, WI) and e.soft (Siemens Medical Solutions, Malvern, PA)) was also investigated to validate the compatibility of the output image on these image analysis and interpretation workstations.

### C. Patient studies

A total of six whole‐body PET/CT scans were acquired on a GE Discovery RX or STE PET/CT scanner^(^ ^4^ ^,^ ^5^ ^)^ in two body segments. The two body segments had an area of overlap that minimized any possible anatomical truncation. In all patient studies, the overlap areas occurred at the level of the lower extremities (legs) below the knee. Three BBs were attached on each leg of the patient just below the knee before the two segment scans were acquired. To ensure the BBs locations are placed in the overlap region between the two PET/CT body segments prior to imaging, the BBs were placed in a fixed location below the knee and the overlap region was then chosen to match this prospective position. PET/CT data acquisition and image reconstruction were performed using the same parameters as in the phantom study.

The software tool was then used to stitch the two PET/CT segments of these patient studies. Evaluation of the software's performance was based on visual inspection, as well as the ability to display the resultant DICOM image set on two different workstations.

## III. RESULTS

### A. Phantom study

**Figure 4:**
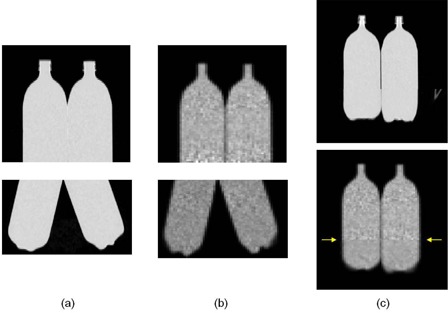
The phantom images in coronal view: (a) CT images of the two segments; (b) PET images of the two segments; (c) stitched PET/CT image.

**Figure 5:**
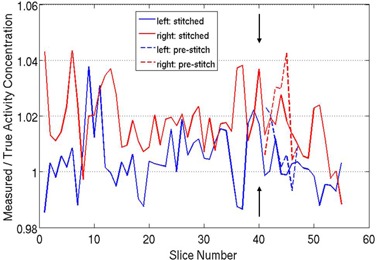
The activity concentration of each axial slice in the stitched PET image. Left and right correspond to the two bottles, respectively, shown in Fig. 4.

Figure 4 shows coronal PET/CT images of the two segments of the phantom, as well as the final stitched PET and CT images. The figure clearly shows that the bottles were re‐orientated between the first and second imaging segments. Visual inspection of Fig. 4(c) shows that the software tool properly stitched the two segments to one another with little mismatch at the stitching plane. A change in image smoothness/texture, however, is seen at the stitching site (arrows) and is addressed in the Discussion section below. Figure 5 shows a plot of the activity concentration along the axial direction of the stitched PET image (solid lines), as well as the activity concentration in the overlap area from the prestitched image (dashed lines). On this plot, slice number 40 (arrows) represents the location of the stitching plane. The plot clearly shows that following decay correction, the activity concentration in the stitched slices is within 5% of the true value. Furthermore, the differences in the activity concentration between prestitched and stitched image (dashed vs. solid lines) are very small. The mismatch between the left and right line profile in Fig. 5 is caused by the slightly different activity concentration between the two bottles. We injected the two bottles separately, which resulted in a slight difference in their activity concentration.

### B. Patient studies

**Figure 6:**
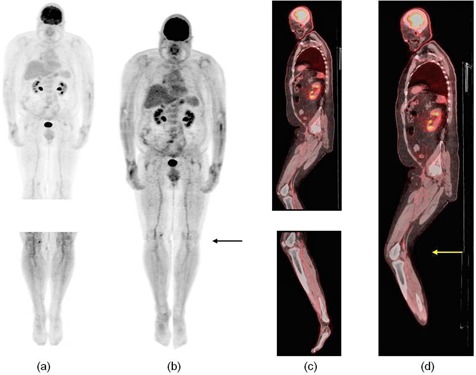
Patient 1: PET MIP images of (a) two body segments and (b) stitched image; fused PET/CT images of (c) two body segments and (d) stitched image.

**Figure 7:**
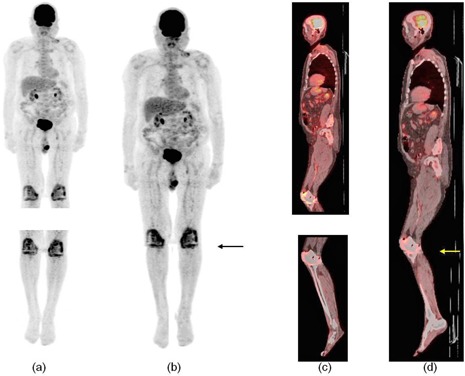
Patient 2: PET images of (a) two body segments and (b) stitched image; fused PET/CT images of (c) two body segments and (d) stitched image.

Figure 6 shows the PET and fused PET/CT images before and after stitching of one patient study. Similar images for another patient are shown in Fig. 7. The stitching site of these two patients was at the level of the lower legs (arrows) and little discontinuity can be seen on the stitched images. All stitched images were able to be viewed on two different commercially‐available PET/CT display workstations (GE Advantage and Siemens e.soft workstations). Both Figs. 6 and 7 are screen captured from a GE Advantage Windows workstation.

## IV. DISCUSSION

This paper describes a software tool that was developed to stitch two PET/CT body segments into a single whole‐body image set. The proposed software tool utilizes simple mathematical techniques to perform this task. In this regard, the value of this tool is in its ability to stitch PET/CT images from two body segments with minimal user interaction, while at the same time facilitating the interpretation of whole‐body PET/CT studies.

Several features are included in the software to facilitate its use: 1) the order of selecting the BBs can be arbitrary; 2) if the user selects the same BB twice, a warning message will pop up indicating that this landmark has already been chosen and asks the user to make another choice; 3) the software can compensate for differences in field‐of‐view sizes and zoom factors between the two segments, as well as between the PET and CT images; 4) the software tool has many user‐friendly utilities such as zoom, window/level adjustment, three orthogonal views (axial, coronal, and sagittal) built within the GUI. Two executable versions of this software tool have been generated for Windows (*.exe) and Linux (*.bin) operating systems, respectively.

The performance of this software tool has been validated using a phantom study and six patient studies. Figure 4(c) showed very little discontinuity in the stitched PET/CT images in coronal view of the phantom study. Since the pixel sizes in CT images are not isotropic (0.97*0.97*3.27 mm), the interpolation of the anisotropic CT pixel may cause some degradation in coronal and sagittal views. This issue can be seen in the stitched CT image in Fig. 4(c), whereby the width of the bottle was slightly widened in the stitched part. The measurement of the activity concentration in Fig. 5 showed the ability of the developed software to perform decay correction. The variation in the activity profiles are within 5% of the true activity in place in the bottles, which is within the expected variability in PET image.

Figures 6 and 7 show the software's ability to stitch PET/CT images of patient studies with little discontinuity or artifacts. Figure 6(b) is a MIP representation of the two image sets after stitching. The difference in activity profiles between the two image sets in the blood vessel might be caused by the difference in circulating blood activity between the two image sets. This difference cannot be accounted for with decay correction, since it is due to physiological difference in biodistribution. Furthermore, a leg–leg collision may occur after stitching the two segments, especially for very large patients. Figures 6(b) and 7(b) show such partial collisions. However, we anticipate that patients will be asked to position their legs apart to minimize the occurrence of such collisions. Finally, the stitched image set is compatible with the GE and Siemens PET/CT display workstations.

One potential improvement of the software lies in the interpolation method that was used to find the value of a voxel in Eq. (2). When solving Eq. (2), the resulting position of voxel most probably did not coincide with a single voxel. Therefore, the value at position needed to be interpolated from several surrounding voxels. Tri‐linear interpolation was applied in the current software version leading to a tradeoff between image quality and operating speed. One significant effect of using interpolation is that it results in image blurring, as shown in Fig. 4(c), where a change in image smoothness at the stitching plane can be seen in the PET image. This blurring effect is equivalent to applying a low pass filter to the original image data. Although blurring is unavoidable in interpolation, one technique to equalize the extent of blurring in both segments is to also blur the upper segment by an equivalent amount, or to de‐blur the images in the lower segment. Both of these approaches, however, will impact the resultant quantitative measurements.

Another potential software improvement is in the method used to determine the location of the BBs. The current software requires the user to manually select the BBs on the CT images. The software tool has the ability to allow users to zoom in to pick the voxel that has the highest CT number. This process may be error‐prone, since a BB is very small and the CT voxel size is approximately $0.97*0.97*3.27\;{\text{mm}}^{3}$; therefore, the error is rather small. The selecting process can be made more convenient, by automatically distinguishing the BBs in the CT image. The main difficulty with such an automated process, however, is that some patients might have metal implants in their legs, such as the patient shown in Fig. 7 (implant in knee). The value of the BBs and the metal are relatively the same in the CT image so that using a simple threshold to identify the BBs location would include the implanted metal as well. One method to overcome this drawback is to select the BBs based on the size of the segmented area. The BBs are only visible on several continuous pixels, while the metal implant usually occupies a larger area. The factor of size, therefore, could be used to help segment the BB from the metal implants. Another potential method that could be used to differentiate the BBs from the metal implants is based on the different positions between the BBs and the metal implants. The BBs are attached to the patient's skin, whereas the metal implants are usually located deeper in the patient's body. In this instance, the software can be designed to search only the surface of the patient leg to locate the BBs.

The stitching approach is based on three points which might result in discontinuity at the stitching site (Figs. 6(b) and 7(b)) because calf/thigh region might deform between the upper and bottom image sets. This problem, however, should not impact patient management unless the discontinuity occurs at the level of tumor. In an effort to avoid this, the end of first and the beginning of second segment will be selected such that no tumor falls in that location, if knowledge of the lesion locations is known a priori. On the other hand, deformable registration has the ability to suppress discontinuities at the stitching site, but it might also affect SUV measurement (due to deforming), which could potentially affect diagnosis. Furthermore, PET images are characterized by low resolution and relatively high noise, which could affect the accuracy of deformable registration. Moreover, since the overlap region between the two image sets is rather small, image registration might not be possible. Additional experiments are warranted to evaluate the feasibility of deformable registration.

This software tool was primarily developed for nuclear medicine PET/CT imaging. However, it can also be used with other imaging modalities such as single photon emission computed tomography/computed tomography (SPECT/CT).

## V. CONCLUSIONS

The developed software tool allows the stitching of two segments of a whole‐body PET/CT scan with minimal user interaction. Such an approach facilitates the interpretation of whole‐body PET/CT scans from a number of scanners with limited extent of bed travel.
